# Increased Endogenous Activity of the Renin-Angiotensin System Reduces Infarct Size in the Rats with Early Angiotensin II-dependent Hypertension which Survive the Acute Ischemia/Reperfusion Injury

**DOI:** 10.3389/fphar.2021.679060

**Published:** 2021-05-28

**Authors:** Zuzana Husková, Soňa Kikerlová, Janusz Sadowski, Petra Alánová, Lenka Sedláková, František Papoušek, Jan Neckář

**Affiliations:** ^1^ Center of Experimental Medicine, Institute for Clinical and Experimental Medicine, Prague, Czechia; ^2^ Department of Renal and Body Fluid Physiology, Mossakowski Medical Research Institute, Polish Academy of Science, Warsaw, Poland; ^3^ Laboratory of Developmental Cardiology, Institute of Physiology of the Czech Academy of Sciences, Prague, Czechia

**Keywords:** renin-angiotensin system, ischemia/reperfusion injury, hypertension, angiotensin II receptor antagonist, infarct size

## Abstract

We investigated the role of the interaction between hypertension and the renin-angiotensin system in the pathophysiology of myocardial ischemia/reperfusion injury. We hypothesized that in the early phase of angiotensin II (ANG II)-dependent hypertension with developed left ventricular hypertrophy, cardioprotective mechanism(s) are fully activated. The experiments were performed in transgenic rats with inducible hypertension, noninduced rats served as controls. The early phase of ANG II-dependent hypertension was induced by five-days (5 days) dietary indole-3-carbinol administration. Cardiac hypertrophy, ANG II and ANG 1–7 levels, protein expression of their receptors and enzymes were determined. Separate groups were subjected to acute myocardial ischemia/reperfusion injury, and infarct size and ventricular arrhythmias were assessed. Induced rats developed marked cardiac hypertrophy accompanied by elevated ANG levels. Ischemia/reperfusion mortality was significantly higher in induced than noninduced rats (52.1 and 25%, respectively). The blockade of AT1 receptors with losartan significantly increased survival rate in both groups. Myocardial infarct size was significantly reduced after 5 days induction (by 11%), without changes after losartan treatment. In conclusion, we confirmed improved cardiac tolerance to ischemia/reperfusion injury in hypertensive cardiohypertrophied rats and found that activation of AT1 receptors by locally produced ANG II in the heart was not the mechanism underlying infarct size reduction.

## Introduction

Despite the progress in understanding the mechanisms and treatment options for cardiovascular diseases (CVDs), ischemic heart disease (IHD) is among the major causes of morbidity and mortality worldwide. In 2017, the population of patients with IHD was estimated at 126.5 million worldwide, with a higher prevalence among males compared to females (68.5 vs. 57.9 million). Between the years 1990 and 2017, the number of people with IHD worldwide increased by 74.9% (Virani et al., 2020).

Early reperfusion of ischemic myocardium is the most effective strategy to reduce infarct size and improve the clinical outcome. This therapy has become a “golden standard” in the treatment since the 1970s (Ginks et al., 1972; Maroko et al., 1972). On the other hand, restoration of coronary artery blood flow may cause cardiomyocyte death and microvascular dysfunction and worsen tissue damage; this phenomenon is termed “myocardial ischemia/reperfusion (I/R) injury” (Sanada et al., 2011; Jennings, 2013). The exact mechanisms of I/R injury are not fully understood but it may paradoxically diminish the beneficial effects of myocardial reperfusion. Thus, reperfusion itself leads *per se* to accelerated and additional myocardial injury beyond that generated by the primary ischemia alone (Bulluck et al., 2016).

After restoring blood flow to ischemic tissue, cells release several chemical mediators to activate reduced nicotinamide adenine dinucleotide phosphate (NADPH) oxidase, such as phospholipase A2, tumor necrosis factor-alpha (TNF-α), interleukin-1beta (IL-1β), interferon-gamma (IFN-γ), and angiotensin II (ANG II). ANG II stimulates its local receptors to increase the expression of NADPH oxidase, resulting in I/R injury (Oudot et al., 2003; Wu et al., 2013; Granger and Kvietys 2015). Both ANG II receptor subtypes are expressed in the heart.

Based on the Framingham cohort study, hypertension and hypertension-induced left ventricular hypertrophy (LVH) were recognized as independent risk factors in the development of CVDs (Vidt and Michael Prisant 2005; Andersson et al., 2019). For many decades it has been accepted that myocardial LVH exacerbates I/R heart damage (Anderson et al., 1987; Dellsperger et al., 1988; Garcia-Dorado et al., 1988; Ferdinandy et al., 2007; Mozaffari et al., 2013). However, some studies did not clearly show increased infarct size in the hypertrophied hearts of hypertensive animals (Matsuhisa et al., 2008; Wagner et al., 2013), and others showed reduced infarct size in hypertensive animals with LVH compared to normotensive animals (Mozaffari and Schaffer, 2003; Neckár et al., 2012; Alánová et al.,). These findings have long been ignored, and the evidence that hypertrophied hearts undergo a greater degree of myocardial damage during I/R injury is not unequivocal.

The renin-angiotensin system (RAS) plays a key role in blood pressure regulation and fluid homeostasis, with an influence on organs and functions throughout the body. The main effector peptides of the RAS are ANG II (vasoconstrictive axis) and ANG 1–7 (vasodilatory axis). A lack of equilibrium between these two axes is associated with cardiovascular disease, including heart failure and chronic kidney disease. The systemic or circulating RAS requires interaction of multiple organs involving liver production of angiotensinogen which is a substrate for generation of ANG I by renin, a protease produced by juxtaglomerular afferent arteriole cells. This step is followed by a second cleavage, to Ang II, exerted by ACE located in lung endothelium. Distinct from the systemic RAS, production of multiple RAS components has been detected in a variety of organs and tissues (local RASs), such as the pancreas, liver, intestine, heart, kidney, vasculature, carotid body, and adipose tissue, as well as in the cells of the nervous, reproductive, and digestive systems. The effects of the local release of RAS paracrine active agents can be differentiated from the endocrine actions (Paul et al, 2006; Leung, 2010; Yang and Xu, 2017). It appears that local and systemic RAS systems act in concert to result in integrated ANG-mediated effects. The actions of ANG II are mediated predominantly by AT1 and AT2 receptors. Therefore, effective treatment strategy in the clinic is to reduce ANG II generation (using ACE inhibitors) or inhibit its actions using AT receptor blockers.

It was recently suggested that hypertension may play a dual role in myocardial I/R injury (Červenka et al., 2018). In the early stage of hypertension and LVH progression, cardioprotective mechanism(s) may be activated. On the other hand, in the late phase of hypertension with already developed signs of end-organ damage the cardiac tolerance to I/R injury could be impaired. Therefore, we decided to examine the role of the interaction between hypertension and the renin-angiotensin system in the pathophysiology of myocardial I/R injury. To obtain a better insight into the role of the RAS activity and its impact on the state of the renal and cardiac function, the results in noninduced (NI) and indol-3-carbinol (I3C)-induced rats were compared between rats treated with losartan (Lozap), a specific ANG II AT1 receptor antagonist, and their untreated counterparts.

Recently, inbred transgenic rats with inducible hypertension [strain name, TGR (*Cyp1a1–Ren-2*)] have been generated using the cytochrome P-450 promoter, *Cyp1a1*, to regulate the expression of the mouse *Ren-2* renin gene (Kantachuvesiri et al., 2001). The renin transgene is rapidly expressed primarily in the liver after exposure to natural xenobiotics such as I3C which can be easily administered with diet. Such enhanced expression of renin transgene results in an increase in ANG II levels and in the development of ANG II-dependent form of hypertension; the transgene expression and hypertension can be controlled in a dose-dependent and reversible manner. In conclusion, the model can be regarded as optimal for studying the interaction between RAS, hypertension and hypertension-induced LVH.

## Materials and Methods

### Ethical Approval, Animals, and Chemicals

The animal experiments were approved by the Animal Care and Use Committee of the Institute for Clinical and Experimental Medicine (IKEM), Prague, consequently, by the Ministry of Health of the Czech Republic (project decision MZDR 32747/2017–3/OVZ), in accordance with the guidelines and practices established by the European Convention on Animal Protection and Guidelines on Research Animal Use, as described in the Directive 2010/63/EU as amended by Regulation (EU) 2019/1010 (http://data.europa.eu/eli/dir/2010/63/2019-06-26).

Experiments were performed in male *Cyp1a1-Ren-2* transgenic rats, at initial age of 3 months and weight of 300 g. In this inbred strain of transgenic rats the degree of ANG II-dependent hypertension, gene expression and the level of endogenously produced ANG II can be precisely controlled in a dose- and time-dependent way (Husková et al., 2010; Honetschlägerová et al.,; Sporková et al., 2014; Husková et al., 2016; Jíchová et al.,). The animals were bred and housed at the Center of Experimental Medicine, IKEM, from stock animals supplied by Professor Mullins from the Center for Cardiovascular Science, University of Edinburgh, United Kingdom. Animals were housed under standard conditions (temperature 22 ± 1°C; relative humidity 40%, 12-h light/dark cycle), fed standard pellet rat chow or rat chow containing 0.3% I3C, and given tap water *ad libitum* or losartan in drinking water. The rats were fasted on the day before the start of I3C-induction to ensure they begin to eat immediately after rat chow with I3C was given.

*Losartan* (Lozap, Zentiva a.s., Hlohovec, Slovak Republic), a specific AT1 receptor antagonist, was given in drinking water at the concentration of 100 mg/L, starting 24 h before the onset of I3C-induction to ensure complete blockade of AT1 receptors, and continued throughout I3C-induction.

### Experimental Design—Series 1

At the end of experiments, all rats were decapitated to collect blood and tissue samples for analysis. The ratio of LVW to tibia length was used to evaluate the degree of left ventricle hypertrophy. The measurements were performed in noninduced rats, and the rats after 12 h (12 h) or five days (5 days) of I3C-induction, losartan treated or untreated. The following experimental groups were examined:

1. noninduced rats/untreated (*n* = 8)
2. noninduced rats/losartan (*n* = 9)
3. 12 h I3C-induced/untreated (*n* = 11)
4. 12 h I3C-induced/losartan (*n* = 9)
5. 5 days I3C-induced/untreated (*n* = 13)
6. 5 days I3C-induced/losartan (*n* = 9)

Plasma and tissue ANG II and ANG 1–7 levels were measured by competitive radioimmunoassay (RIA) using the commercially available (ED29051, IBL International, Hamburg, Germany) and custom-made (Immunotech s.r.o., Prague, Czech Republic) RIA kits, respectively. Briefly, immediately after the decapitation, whole blood was collected into cold tube containing inhibitor cocktail (5 mM ethylendiamintetraacetic acid (EDTA), 1.25 mM 1,10-phenanthroline, 20 μM enalapril maleate, 10 μM pepstatin A), kidneys and heart were removed, surface dried using gauze swabs, weighed and 0.5 g of the tissue was homogenized in 3 ml precooled methanol and centrifuged. 1 ml of plasma was precipitated with 4 ml of ethanol and again centrifuged. Supernatants were evaporated using a vacuum centrifuge. In addition, kidney samples were purified by solid-phase extraction and evaporated again. Dried plasma and kidney samples were stored at −20°C or lower until assayed (for detailed methods see Supplementary Material).

Protein expression of AT1 and Mas receptors, of ACE and ACE2 was quantified in kidney cortex by Western-blot analysis (see Supplementary Material, which include all detailed methods and the antibodies used). All protein data was normalized to the housekeeping protein β-actin.

### Experimental Design—Series 2

Separate experimental groups of rats, as in series 1 (*n* = 10–23, in each group), were subjected to regional myocardial I/R as described previously, using an open-chest model (Neckár et al., 2012; Alánová et al.,; Hrdlička et al., 2019). Anesthetized (pentobarbital sodium, 60 mg/kg i.p.) intubated rats were ventilated with room air at 68 strokes/min (Ugo Basile, Italy; tidal volume of 1.2 ml/100 g body weight), the rectal temperature was maintained between 36.5 and 37.5°C using a heating pad throughout the experiment. The left anterior descending (LAD) coronary artery occlusion was performed. Characteristic changes in myocardial color and the incidence of ischemic arrhythmias verified the complete coronary artery occlusion. After 20 min of regional ischemia, reperfusion of previously ischemic tissue continued for 3 h. At the end of reperfusion, the heart was excised and washed with saline *via* aorta. The infarct size and the area at risk were determined by staining with potassium permanganate and 2,3,5-triphenyltetrazolium chloride (Sigma Aldrich, Prague, Czech Republic), respectively. The infarct size was normalized to the area at risk. This approach has been validated in many experimental studies and is currently accepted as the “gold standard” for measurement of myocardial infarct size in small animals (Sanada et al., 2011). The incidence and severity of ventricular arrhythmias during the 20-min ischemia and first 3 min of reperfusion were assessed according to the Lambeth Conventions (Walker et al., 1988). Premature ventricle complexes (PVCs) occurring as singles, salvos (2 or 3 PVCs) or tachycardia (VT, a run of 4 or more consecutive PVCs) were counted separately. The incidence and duration of life-threatening ventricular tachyarrhythmias, i.e., VT and fibrillation (VF), were also determined. VF lasting more than 2 min was considered as sustained (sVF). Rat hearts exhibiting VFs were excluded from further evaluation, such hearts are exposed to a tremendous ischemic insult that further alters the infarct size. However, the data were used for analysis of incidence and severity of arrhythmias. For detailed methods see Supplementary Material.

### Statistical Analysis

Data are expressed as means ± SEM. Graph-Pad Prism software (Graph Pad Software, San Diego, California, United States) was used for statistical analysis. Multiple-group comparisons were performed by regular two-way analysis of variance followed by Tukey’s *post hoc* test as appropriate. The incidence of tachycardia and fibrillation was examined by Fischer’s exact test. One-way ANOVA or ANOVA for repeated measurements with Student-Neumann-Keuls test was performed in normally distributed variables. Differences in arrhythmias among the groups were compared by the Kruskal–Wallis nonparametric test. Values exceeding the 95% probability limits (*p* < 0.05) were considered statistically significant. The data that were not normally distributed (arrhythmias) were expressed as median_interquartile range.

## Results

### Series 1: Kidney Studies

**FIGURE 1:**
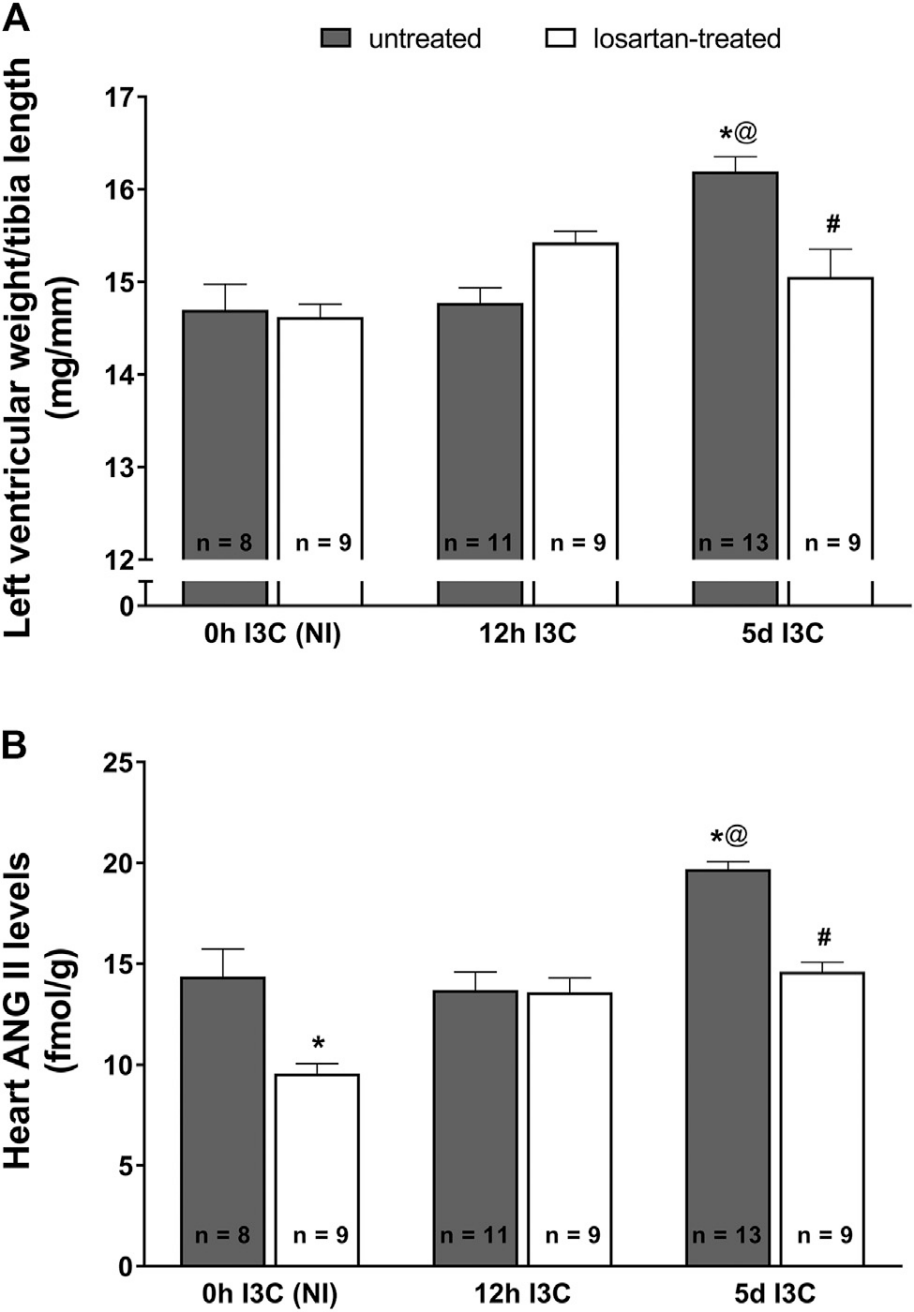
The ratio of left ventricle weight to tibia length **(A)** and heart angiotensin II (ANG II) levels **(B)** in untreated and losartan-treated noninduced (NI) and I3C-induced *Cyp1a1-Ren-2* transgenic rats. Values are expressed as means ± SEM. *p < 0.05 vs. untreated noninduced rats; ^#^ *p* < 0.05 vs. untreated group at the same time point; ^@^ *p* < 0.05 vs. untreated 12 h I3C-induced rats. Multiple-group comparisons were performed by regular two-way analysis of variance followed by Tukey‘s post hoc test.

The ratio of left ventricular weight (LVW) to tibia length was used to determine the degree of cardiac hypertrophy. As shown in Figure 1A, after 5 days (5 days) of I3C-induction untreated rats developed marked cardiac hypertrophy in comparison with the noninduced (NI) and those subjected to 12 h I3C-induction (16.2 ± 0.2 vs. 14.7 ± 0.3 and 14.8 ± 0.2 mg/mm). This effect was attenuated by losartan treatment (15.1 ± 0.3 vs. 16.2 ± 0.2%). The cardiac hypertrophy was accompanied by elevated heart ANG II level (19.7 ± 0.4 vs. 14.4 ± 1.4 fmol/g) that was lowered by losartan treatment as observed in NI rats (Figure 1B).

**FIGURE 2:**
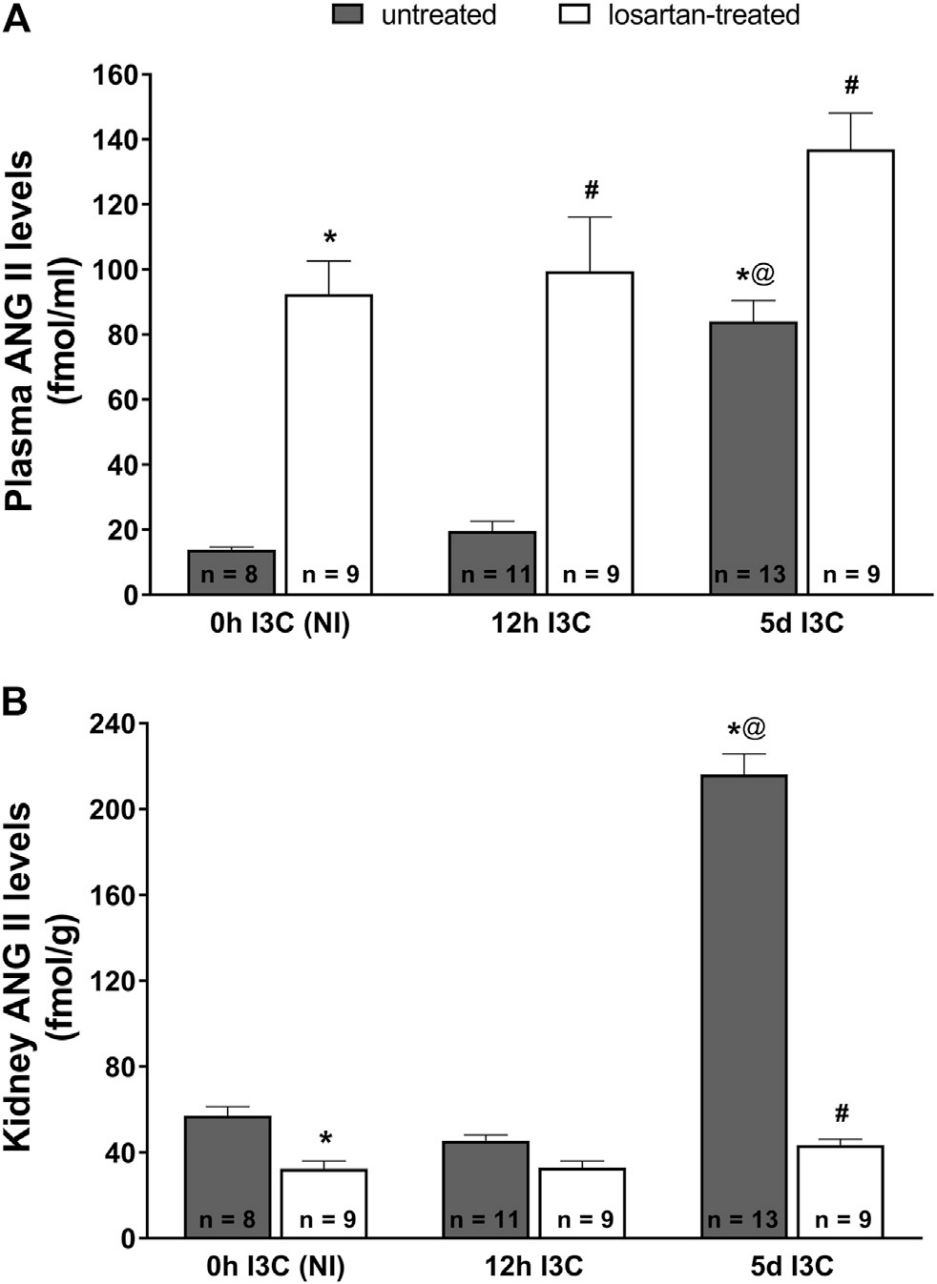
Plasma angiotensin II (ANG II) levels **(A)** and kidney ANG II levels **(B)** in untreated and losartan-treated noninduced (NI) and I3C-induced *Cyp1a1-Ren-2* transgenic rats. Values are expressed as means ± SEM. *p < 0.05 vs. untreated noninduced rats; ^#^ *p* < 0.05 vs. untreated group at the same time point; ^@^ *p* < 0.05 vs. untreated 12 h I3C-induced rats. Multiple-group comparisons were performed by regular two-way analysis of variance followed by Tukey’s post hoc test.

Five-days’ I3C-induction resulted in a significant 6-fold rise of plasma ANG II level (84 ± 6.4 vs. 13.9 ± 0.7 fmol/ml) (Figure 2A). Losartan treatment increased plasma concentrations of ANG II irrespective of the induction durection applied. 5 days I3C-induction also resulted in about 4-fold higher kidney ANG II level in comparison to NI and 12 h I3C-induced rats (216.1 ± 9.6 vs. 57.1 ± 4.2 and 45.6 ± 2.6 fmol/g) and this effect was reduced by losartan treatment to the level observed in NI rats (Figure 2B).

**FIGURE 3:**
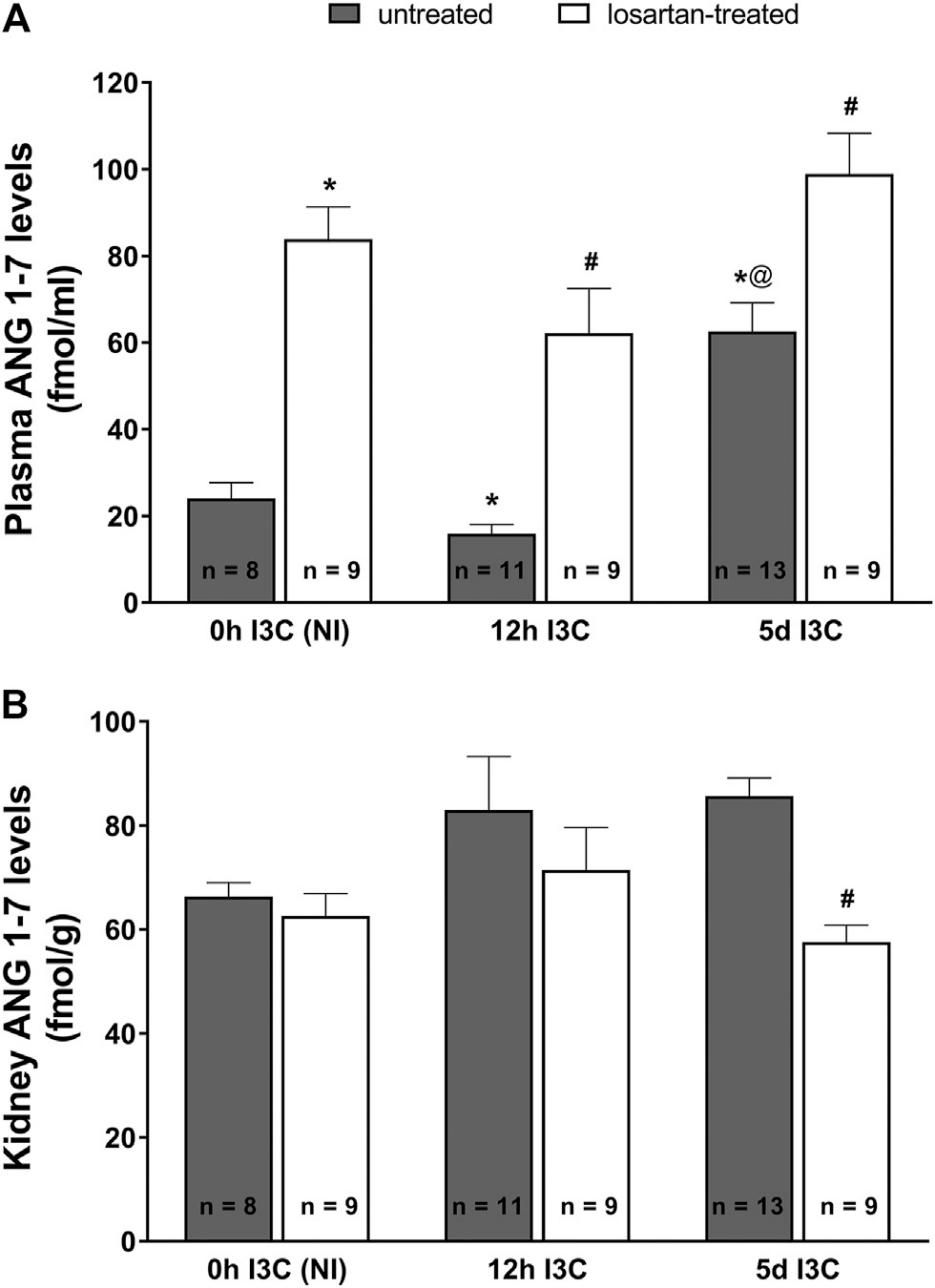
Plasma angiotensin 1–7 (ANG 1–7) levels **(A)** and kidney ANG 1–7 levels **(B)** in untreated and losartan-treated noninduced (NI) and I3C-induced *Cyp1a1-Ren-2* transgenic rats. Values are expressed as means ± SEM. *p < 0.05 vs. untreated noninduced rats; ^#^ *p* < 0.05 vs. untreated group at the same time point; ^@^ *p* < 0.05 vs. untreated 12 h I3C-induced rats. Multiple-group comparisons were performed by regular two-way analysis of variance followed by Tukey’s post hoc test.

As shown in Figure 3A, the changes in plasma angiotensin 1–7 (ANG 1–7) levels displayed a profile similar as that for plasma ANG II levels. There were no differences in kidney ANG 1–7 levels between NI and induced rats throughout the experiment (Figure 3B). Losartan treatment significantly decreased renal concentration of ANG 1–7 in 5 days I3C-induced rats (57.6 ± 3.3 vs. 85.6 ± 3.5 fmol/g).

**FIGURE 4:**
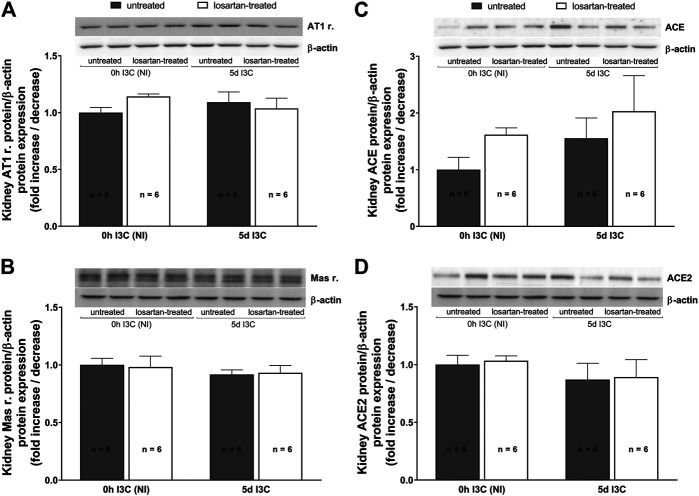
Protein expressions of renal AT1 **(A)** and Mas **(B)** receptors, ACE **(C)** and ACE2 **(D)** in untreated and losartan-treated noninduced (NI) and I3C-induced *Cyp1a1-Ren-2* transgenic rats. Values are expressed as means ± SEM. Multiple-group comparisons were performed by regular two-way analysis of variance followed by Tukey’s post hoc test.

Protein expressions of renal AT1 and Mas receptors, ACE and ACE2 did not significantly differ between induced and NI, treated and untreated rats (Figures 4A–D).

### Series 2: Heart Studies

The results of Series 1 and also our earlier results indicated that after 5 days I3C-induction of renin gene, *Cyp1a1-Ren-2* transgenic rats showed markedly elevated blood pressure (BP) approaching a plateau, and clearly developed LVH without signs of cardiac decompensation (Husková et al., 2010). Therefore we decided to compare the cardiac ischemic tolerance in 5 days I3C-induced (i.e., in the early phase of ANG II-dependent hypertension) and in NI rats.

**FIGURE 5:**
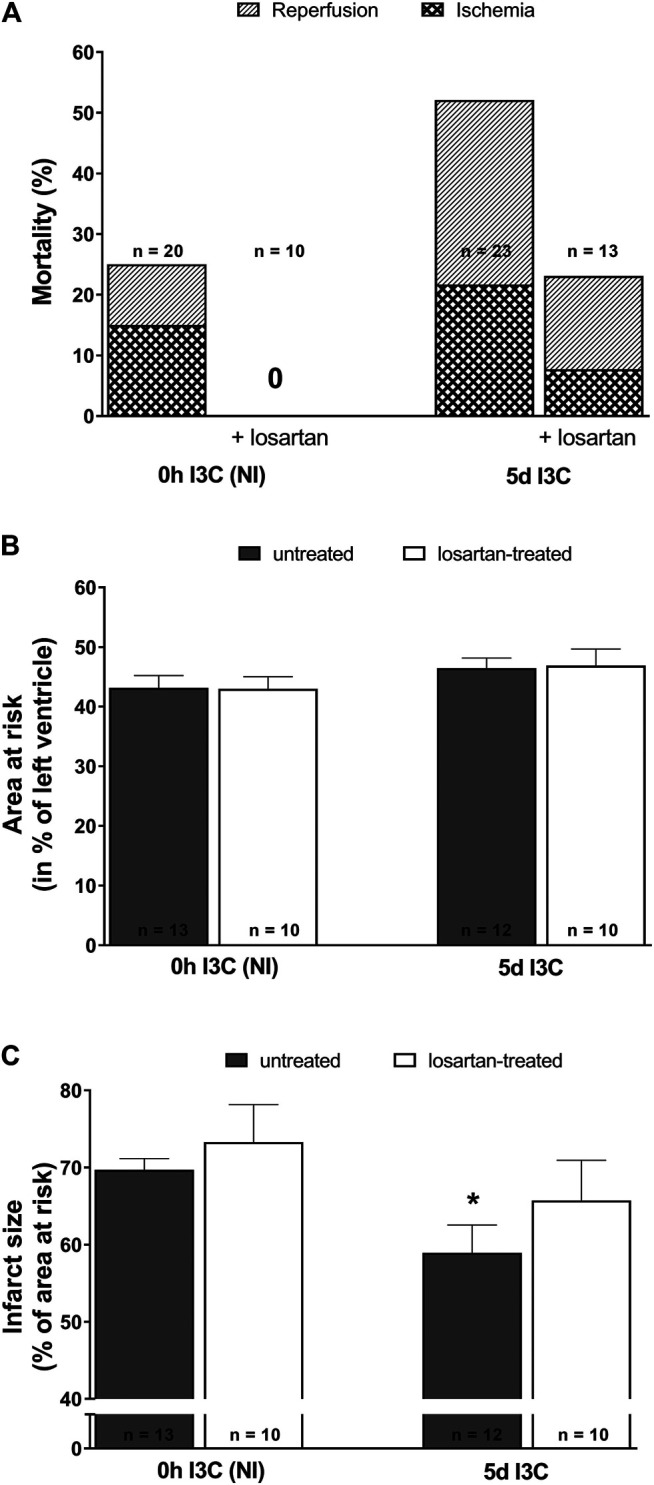
Mortality **(A)**, myocardial area at risk normalized to the size of the left heart ventricle **(B)** and infarct size presented as per cent of the area at risk **(C)** in untreated and losartan-treated noninduced (NI) and I3C-induced *Cyp1a1-Ren-2* transgenic rats. Values are expressed as means ± SEM. *p < 0.05 vs. untreated noninduced rats. Data were analyzed by Fischer’s exact test (mortality) and by One-way ANOVA with Student-Neumann-Keuls test (relative size of the area at risk and infarct size).

The acute ischemic and early reperfusion mortality reached 52% in 5 days I3C-induced and 25% in NI rats (Figure 5A). Losartan treatment decreased mortality in both 5 days I3C-induced (to 23.1%) and NI group (down to 0%). The mean area at risk (normalized to the size of the LV) was 43–47% and did not significantly differ among groups (Figure 5B). The myocardial infarct size (normalized to the area at risk) was 69.7 ± 1.5% in untreated NI rats (Figure 5C). Five-days I3C-induction significantly reduced myocardial infarction size to 58.9 ± 3.6%. This pattern was not changed by losartan treatment, i.e., myocardial infarction decreased from 73.3 ± 4.9% in treated NI to 65.7 ± 5.2 in treated 5 days I3C-induced rats. However, the difference between the untreated vs. treated NI and untreated vs. treated 5 days I3C-induced groups did not reach statistical significance. Representative images of myocardial infarction induced by 20-min coronary artery occlusion and 3-h reperfusion in individual experimental groups are shown in Supplementary Figure S1.

**FIGURE 6:**
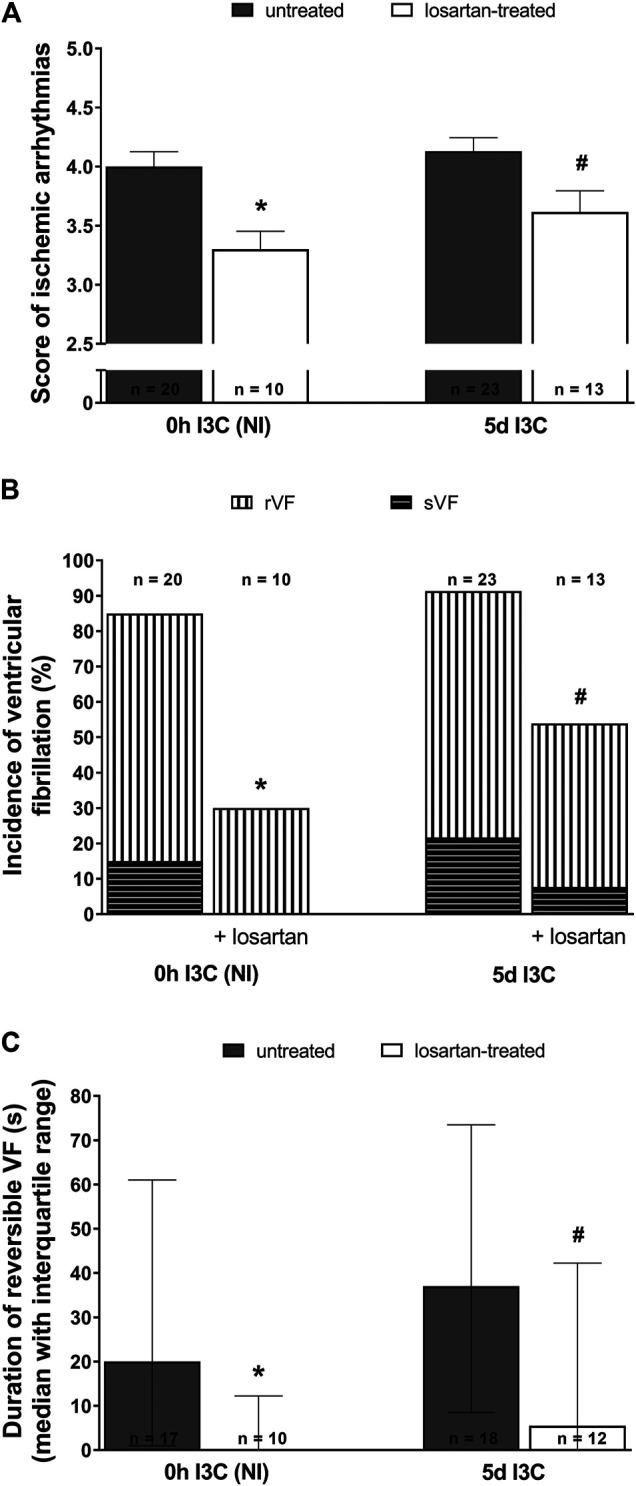
Ischemic arrhythmias: the score of ischemic arrhythmias **(A)**, the incidence of ventricular fibrillation, both reversible and sustained **(B)** and, duration of reversible ventricular fibrillation (VF) **(C)** in untreated and losartan-treated noninduced (NI) and I3C-induced *Cyp1a1-Ren-2* transgenic rats. Values are expressed as means ± SEM or as median with interquartile range. *p < 0.05 vs. untreated noninduced rats; #*p* < 0.05 vs. untreated group at the same time point. Data were analyzed by Fischer’s exact test (incidence of VF) and by one-way ANOVA or ANOVA for repeated measurements with Student-Neumann-Keuls test in normally distributed variables (score of ischemic arrhythmias). Differences in the duration of reversible VF between the groups were compared by the Kruskal–Wallis nonparametric test.

Untreated NI and 5 days I3C-induced groups did not differ regarding their ischemic arrhythmogenesis, as shown in Figure 6; Table 1. Losartan treatment significantly decreased the score of ischemic arrhythmias, the incidence of ventricular fibrillation (VF) and the median of reversible VF duration in both NI and 5 days I3C-induced groups (Figure 6). Similarly, there was a trend toward a reduction in the number of ischemic PVCs and the duration of tachyarrhythmias in losartan-treated groups but it did not reach statistical significance due to high variability of the values (Table 1).

**TABLE 1:** The number of premature ventricle complexes (PVCs) occurring as singles, salvos, ventricular tachycardia (VT), and total number of premature ventricular complexes, duration of VT (VTd), the number of reversible ventricular fibrillation (rVFe) and VT episodes (VTe) during 20-min coronary artery occlusion in untreated and losartan-treated noninduced (NI) and I3C-induced *Cyp1a1-Ren-2* transgenic rats. Representative electrocardiograms are shown in Supplementary Figure S2.

	0 h I3C (NI)		5 days I3C	
	Untreated	Losartan-treated	Untreated	Losartan-treated
Singles	157 (253)	108 (212)	144 (410)	128 (121)
Salvos	78 (80)	40 (113)	71 (243)	51 (75)
VT	1,200 (2,631)	501 (2011)	1,214 (3,718)	486 (2,665)
PVCs	1,572 (2,746)	776 (2003)	1,462 (3,807)	588 (2,812)
VTe	34 (67)	27 (42)	39 (88)	23 (53)
VTd (s)	100 (212)	46 (166)	99 (281)	52 (196)
rVFe	1 (8)	0 (3)	4 (7)	1 (3)

Values are expressed as medians (range). Data were analyzed by one-way ANOVA or ANOVA for repeated measurements with the Kruskal–Wallis nonparametric test.

**FIGURE 7:**
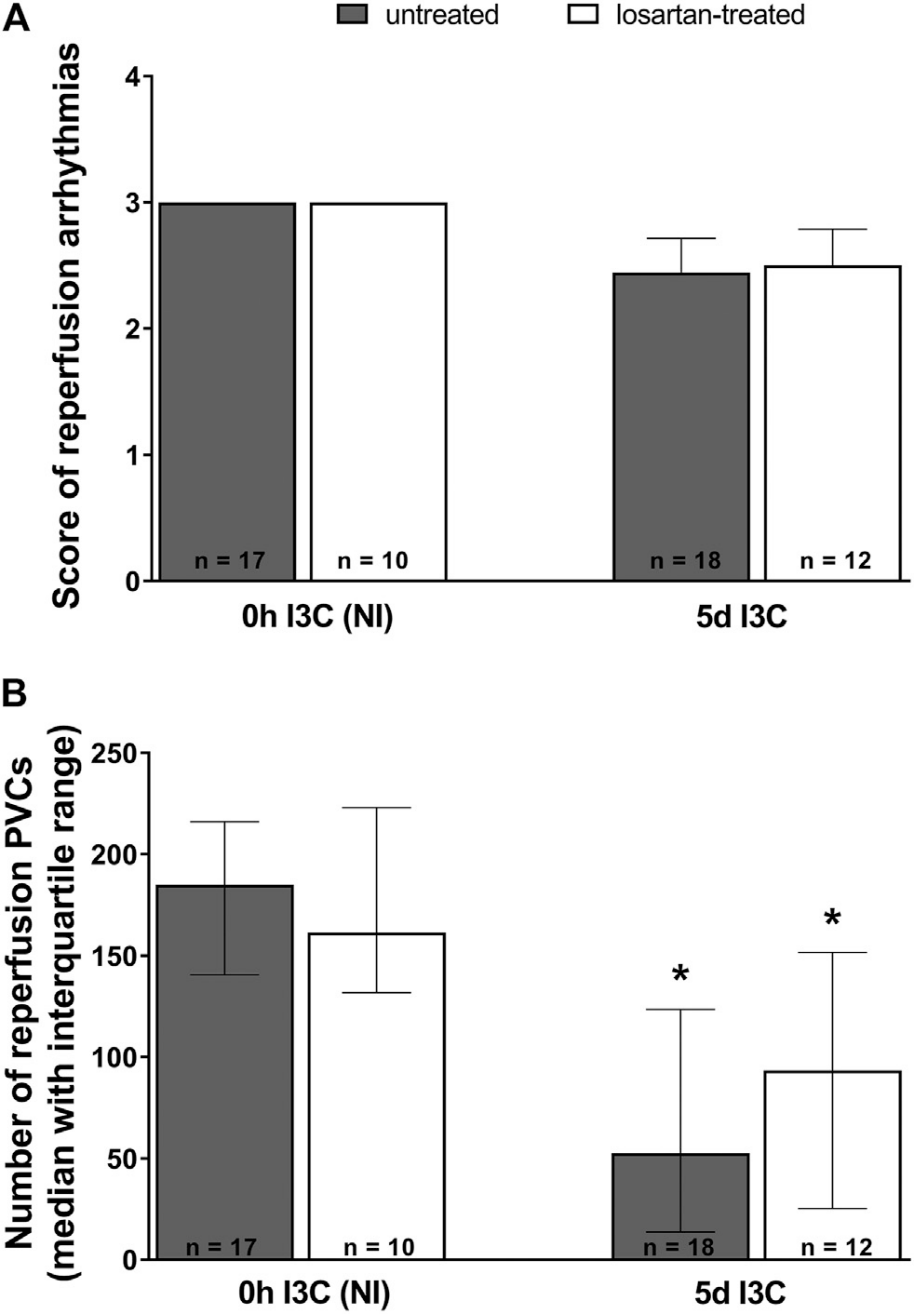
Reperfusion arrhythmias: the score of reperfusion arrhythmias **(A)** and the number of reperfusion premature ventricular complexes (PVCs) **(B)** in untreated and losartan-treated noninduced (NI) and I3C-induced *Cyp1a1-Ren-2* transgenic rats. Values are expressed as means ± SEM or as a median with interquartile range. *p < 0.05 vs. untreated noninduced rats. Data were analyzed by one-way ANOVA or ANOVA for repeated measurements with Student-Neumann-Keuls test in normally distributed variables (score of reperfusion arrhythmias). Differences in the number of PVCs between the groups were compared by the Kruskal–Wallis nonparametric test.

Neither 5 days I3C-induction nor losartan treatment affected the score of reperfusion arrhythmias (Figure 7A). However, the number of reperfusion PVCs was significantly lower in both 5 days I3C-induced groups compared to the corresponding NI groups (Figure 7B).

## Discussion

The most commonly used animal models of ANG II-dependent hypertension display obvious limitations related to the way hypertension is induced. In addition, the exact onset of hypertension is variable and cannot be precisely controlled. It is very difficult to control the activity of endogenous RAS, e.g., in ANG II-infused rats high BP after chronic infusion of exogenous ANG II leads to unphysiological suppression of the endogenous RAS (Mullins and Mullins 2003; Kobori et al., 2007). BP elevation in two-kidney, one-clip (2K1C) Goldblatt rat hypertension secondary to unilateral renal artery stenosis yields variable degrees of RAS activation (Navar et al., 1998; Pinto et al., 1998). In mouse *Ren-2* renin transgenic rats (TGR), hypertension depends on the constitutive expression of inserted and constantly activated transgenes. To avoid all these limitations, a new inbred *Cyp1a1-Ren-2* transgenic rat strain with precisely controlled hypertension was generated (Kantachuvesiri et al., 2001). We and others have demonstrated that gene expression, the level of endogenously produced ANG II, and the degree of subsequent hypertension can be precisely controlled in a dose and time-dependent manner (Peters et al., 2008; Erbanová et al., 2009; Peters et al., 2009; Husková et al., 2010; Husková et al., 2016; Honetschlägerová et al.,; Peters et al., 2012; Cunningham et al., 2013; Sporková et al., 2014; Jíchová et al.,; Sedláková et al.,). We believe that this model is the optimal choice for studies evaluating the exact nature of the relationship between the activity of endogenous RAS, the phase of hypertension and the degree of cardiac ischemic tolerance.

This study tested our hypothesis that in the early phase of ANG II-dependent hypertension with developed LVH but no signs of cardiac decompensation, the cardioprotective mechanism(s) are fully activated. In case of inappropriate RAS activation, this system becomes a major contributor to the development of ANG II-dependent forms of hypertension. The main finding of this study is that the infarct size was slightly but significantly reduced (by 11%) in 5 days I3C-induced hypertensive rats with LVH, compared to noninduced normotensive rats; this indicated some moderate myocardial resistance to I/R injury. Improved cardiac tolerance to irreversible I/R injury was also observed in 2K1C rats, a model of human renovascular hypertension (Alánová et al.,), and in TGR (Neckár et al., 2012). These findings are in disagreement with the original clinical observation that “hypertrophic myocardium is more vulnerable to I/R than normal myocardium”, as well as the results of studies showing that infarct size is increased in hypertensive rats with LVH (Anderson et al., 1987; Dellsperger et al., 1988; Garcia-Dorado et al., 1988; Ferdinandy et al., 2007; Matsuhisa et al., 2008; Mozaffari et al., 2013; Penna et al., 2014). It is clear that the influence of hypertension on tolerance of the hypertrophied myocardium to I/R injury is still not fully elucidated. In addition, ANG II has been shown to reduce infarct size in a concentration-dependent manner in isolated rat hearts (Ford et al., 2001), and in the present study we confirmed significantly elevated tissue and plasma ANG II levels after 5 days I3C-induction.

We found that *Cyp1a1-Ren-2* transgenic rats showed markedly elevated plasma and tissue levels of ANG II, the main biologically active peptide of the RAS vasoconstrictive axis; these animals clearly developed LVH after 5 days I3C-induction of renin gene, unlike those after 12 h I3C-induction and the noninduced rats. Several studies have shown that activation of the vasodilatory “ACE2/ANG 1–7/Mas receptor” axis, the one that usually counteracts the effects of vasoconstrictive “ACE/ANG II/AT1 receptor” axis and is therefore considered as beneficial, prevents and even attenuates the ANG II-induced damages observed in cardiovascular disease (Shi et al., 2010; Patel et al., 2016). The influence of the RAS depends on the balance between vasoconstrictive and the opposing vasodilatory axis. Accordingly, we found that kidney ANG 1–7 levels were twice as high as the levels of ANG II after 12-h induction. However, during the next 5 days of induction, there was also a significant increase in kidney ANG II levels, reflecting rising activity of the vasoconstrictive axis.

The functions of ANG II in the cardiovascular system are predominantly mediated by AT1 receptors (Widdop et al., 2003; Zhuo and Li, 2011) which activate a number of signaling cascades, and this activation determines the actual diverse physiological effects (Mehta and Griendling, 2007; Dolgacheva et al., 2016). The signalling pathways activated by ANG II fall into two categories: G protein-coupled and not G protein-coupled. In addition, ANG II cross-talks with several tyrosine kinases, including receptor and non-receptor tyrosine kinases, it activates serine/threonine kinases, and NAD(P)H oxidases, leading to the formation of the reactive oxygen species. In patients with hyperactive RAS or those with enhanced responsiveness to ANG II, these pathways may initiate and propagate pathological events promoting vascular disease. AT1 receptor blockers (ARBs), together with ACE inhibitors, belong to the most important drugs in the treatment of CVDs. They inhibit the vasoconstrictive axis of the RAS and thereby reduce the risk of cardiovascular morbidity and mortality, IHD and heart failure. It was also shown that ARBs increase Ang 1–7 expression systemically and locally. Remarkably, the majority of the effects of ARBs resemble those of Ang 1–7, which, indeed, was shown to directly interact with AT1 receptor, leading to its functional down-regulation (Shi et al., 2010). In our study, we confirmed an elevation of plasma levels of both ANG II and ANG 1–7 after losartan treatment, maintained throughout the experiment. The effect of ARB blockade in the tissue was observed after 5 days induction, when the RAS was fully activated. Moreover, there was a clear effect of losartan treatment on tissue ANG II levels in NI rats, which was abolished at the onset of I3C-induction.

The second important set of findings in this study concerns cardiac ischemic tolerance and a possible cardioprotective mechanism in the early phase of ANG II-dependent hypertension and LVH, and the effects of angiotensin II AT1 receptor blockade obtained by losartan treatment. Myocardial I/R injury has not been previously studied in rats with the early phase of ANG II-dependent hypertension. We found that during ischemia and subsquent reperfusion, mortality was more than doubled in the rats after 5 days induction, with higher values in reperfusion, despite reduction of the number of reperfusion PVCs. Losartan treatment resulted, among other effects, in a significant decrease in mortality by reducing the frequency of life-threatening ischemic arrhythmias. These results are consistent with the previously cited studies (Lee et al., 1997; Zhu et al., 2000). Based on our data, we are not able to say whether the treatment with losartan is cardioprotective in hypertensive patients or not. We can only say that in the early phase of hypertension this treatment is antiarrhythmogenic.

The results obtained at different time points of renin gene induction with expected different effects on myocardial ischemic tolerance, might be used in attempts to define the critical therapeutic time window of protection, by analogy with the pattern of delayed ANG II-induced preconditioning. If the expected ischemia-resistant cardiac phenotype of *Cyp1a1-Ren-2* rats in the optimal time window after renin gene induction depends on a mechanism analogous to that of acute preconditioning induced by AT1 receptor activation (Liu et al., 1995; Nakano et al., 1997; Hostrup et al., 2012), it should be abolished by AT1 receptor blockade. We confirmed local activation of RAS in the heart, thus, a reduced number of reperfusion PVCs and reduced infarct size in hypertensive rats with LVH would indicate the protective effects of ANG II in the early phase of hypertension. If such expected beneficial effect of ANG II on infarct size was simply the result of AT1 receptor activation, it should be abolished by AT1 receptor blockade with losartan, and the infarct size should increase. However, after losartan treatment we did not observe any significant changes in these parameters, apart from a slight (increasing) trend in infarct size, which suggested that activation of AT1 receptors by ANG II locally produced in the heart is not the main mechanism underlying infarct size reduction. Similarly, chronic losartan treatment did not change myocardial infarction in hypertensive Dahl salt-sensitive rats (Matsuhisa et al., 2008), moreover, it impaired lethal myocardial injury caused by acute I/R insult in normotensive Sprague-Dawley rats (Song et al., 2015). Based on our present results, the exact mechanism cannot be defined and this is also the case with some other mechanisms. The reduced infarct size in the early phase of hypertension could be caused by heart adaptive responses, such as HIF-1 alpha induction, the action of heat-shock proteins, and induction of cardioprotective adipokines, mitochondria proliferation, etc. Recent reports indicate that Ang II-induced preconditioning exerts cardioprotective effects against I/R injury *via* AT2 receptors. Cardioprotective effects are characterized by improved cardiac function, reduced mitochondrial permeability transition pore opening, improved respiratory control index and reduced infarct size and LDH release (Nuñez et al., 2018). Both receptor subtypes for ANG II exist in the heart. In adult rat cardiomyocytes AT2 receptors are expressed at low levels, however, they could be upregulated in pathophysiological conditions such as hypertension, heart failure, and heart hypertrophy (Lee et al., 1997; Kaschina et al., 2017). The data on mitochondrial Ang II showed that AT2 receptors are cardioprotective through their permissive action on AT1 receptor signaling and the suppression of cardiac function. It has been shown that AT1 receptor blockade with losartan in ANG II preconditioning, which promotes selective AT2 receptor activation, reduces infarct size (Nuñez et al., 2018). Another contributors to improved cardiac tolerance to I/R injury could be the counter-regulatory RAS peptides: Ang 1–7 and Ang 1–9 (Widdop et al., 2003; McKinney et al., 2014).

Besides ANG II, endothelin (ET), a powerful vasoconstrictor, participates in the control of the vascular tone and is also responsible for the complications associated with hypertension. However, we showed that blockade of the ET system does not prevent or attenuate the rapid development of severe hypertension in *Cyp1a1-Ren-2* transgenic rats (iTGR) (Vaňourková et al.,). Nevertheless, given the prevention of the rise in cardiac ET-1 by ET receptor inhibition, the blockade might be used to protect rats from hypertensive cardiac damage. Moreover, we showed that nonselective ET (A/B) receptor blockade markedly improves the survival rate and attenuates end-organ damage in homozygous male TGRs without significantly lowering BP (Dvořák et al., 2004). Furthermore, combined RAS and ET (A) receptor blockade exhibits additive beneficial effects on survival rate and the progression of CKD in Ren-2 transgenic rats (TGR) after 5/6 renal ablation (Čertíková ). The effect of lowering BP by combined ET(A) and RAS blockade in TGR is largely dependent on the effects exerted by RAS blockade, but further effects can be attributed to decreased calcium influx due to chronic ET (A) blockade (Vaněčková et al.,).

It can also be expected that the secretion of natriuretic peptides will be increased in response to myocardial stretch to counteract myocardial fibrosis and hypertrophy, to increase natriuresis and cause vasodilatation. Unfortunately, simultaneous additional monitoring of natriuretic peptides and other systems was not possible for technical reasons.

In summary, we found that the activity of the vasoconstrictive and the opposed vasodilatory axis of RAS changed differently during the development of ANG II-dependent hypertension. We observed a clear effect of AT1 receptor blockade on ANG II levels in both induced and non-induced rats. The mortality during myocardial ischemia and subsequent reperfusion was significantly higher in rats after 5 days I3C-induction and was reduced by AT1 receptor blockade. The infarct size after I/R insult was slightly but significantly reduced in 5 days I3C-induced hypertensive rats with LVH, compared to NI normotensive rats, which suggests moderately increased tolerance of cardiac ischemic However, we showed that activation of AT1 receptors by locally produced ANG II in the heart is not the mechanism underlying infarct size reduction.

## Data Availability

The raw data supporting the conclusions of this article will be made available by the authors, without undue reservation.

## Abbreviations

2K1C rats, two-kidney, one-clip Goldblatt hypertensive rats; ACE, angiotensin-converting enzyme; ACE2, angiotensin-converting enzyme 2; ANG 1–7, angiotensin 1–7; ANG II, angiotensin II; ARBs, AT1 receptor blockers; AT1, ANG II receptor type 1; BP, blood pressure; BW, body weight; CVDs, cardiovascular diseases; EDTA, ethylendiamintetraacetic acid; I3C, indol-3-carbinole; IFN-γ, interferon-gamma; IL-1β, interleukin-1beta; IHD, ischemic heart disease; LAD, left anterior descending coronary artery; LVH, left ventricular hypertrophy; LVW, left ventricular weight; Mas, ANG 1–7 receptor; NADPH, reduced nicotinamide adenine dinucleotide phosphate; NI, noninduced; PVCs, premature ventricular complexes; RAS, renin-angiotensin system; RIA, radioimmunoassay; rVF, reversible ventricular fibrillation; sVF, sustained ventricular fibrillation; TGR, mouse *Ren-2* renin transgenic rats; TNF-α,tumor necrosis factor-alpha; VF, ventricular fibrillation; VT, ventricular tachycardia
