# Supplementary material for: Increased Endogenous Activity of the Renin-Angiotensin System Reduces Infarct Size in the Rats with Early Angiotensin II-dependent Hypertension which Survive the Acute Ischemia/Reperfusion Injury
Source: Front Pharmacol. 2021 May 28;12:679060. doi: 10.3389/fphar.2021.679060 (PMC8193500; doi:10.3389/fphar.2021.679060)
Supplement: Supplementary file 1 [file DataSheet1.docx]

Supplementary Material

**DETAILED METHODS**

**Increased endogenous activity of the renin-angiotensin system reduces infarct size in the rats with early angiotensin II-dependent hypertension which survive the acute ischemia/reperfusion injury.**

Zuzana Husková, Soňa Kikerlová, Petra Alánová, Janusz Sadowski, František Papoušek, Jan Neckář

***Experimental design – SERIES 1***

The ratio of left ventricular weight (LVW) to tibia length was used to determine the degree of cardiac hypertrophy. Based on our and others experiences (1–4), this is the most appropriate index to assess cardiac hypertrophy when the development of hypertension in untreated I3C-induced rats is associated with profound body weight (BW) loss.

**The measurement of plasma and tissue angiotensin II (ANG II) concentrations**

***Preparation of blood samples:*** immediately after decapitation, whole blood was collected into cold tubes containing 250 μl of inhibitor cocktail (5 mM EDTA, 1.25 mM 1,10-phenanthroline,
20 μM enalapril maleate, 10 μM pepstatin A), kept on ice (rocked) and then centrifuged at 4°C and 3000 g for 10 min to separate the plasma. After centrifugation, 1 ml of plasma was precipitated with 4 ml of ethanol and again centrifuged at 4°C and 3000 g for 10 min. The supernatant was evaporated using a vacuum centrifuge (Savant SpeedVac). Dried samples were stored at -20°C or lower until assayed.

***Preparation of kidney and heart samples:*** immediately after decapitation and blood collection, kidneys were removed, dried, weighed and 0.5 g of the tissue was homogenized in 3 ml precooled methanol. Heart was also removed, dried, weighed, divided into atria, left ventricle + septum and right ventricle, and 0.5 g of the left ventricle tissue was homogenized in 3 ml precooled methanol. The tube with homogenate was kept on ice and then centrifuged at 4°C and 3000 g for 10 min. The supernatant was evaporated using Savant SpeedVac vacuum centrifuge. Dried samples were stored at -20°C or lower until solid-phase extraction (in the case of kidney samples) and the assay. Kidney homogenates were purified by solid-phase extraction. Dried kidney samples were reconstituted with 4 ml of 50 mM sodium phosphate buffer (pH 7.4) containing 267 mg bovine serum albumin/l and kept on ice. Phenyl-bonded solid phase extraction columns (SPE) (Bond-Elut^®^PH, Agilent) were preconditioned with methanol (3 ml), followed by distilled-water (2x 3 ml). Thereafter, reconstituted kidney samples were applied to pre-washed columns. The columns were sequentially washed with distilled-water (3 ml), hexane (3 ml) and chloroform (3 ml); water removes salts and other polar substances from the columns. Hexane and chloroform elute contaminating lipids and hydrophobic material from the columns but do not affect angiotensin peptides recovery. At the end, angiotensin peptides were eluted from SPE columns using 2x 1 ml flush of methanol. The eluates were evaporated to dryness using a vacuum centrifuge. Dried samples were stored at -20°C or lower until assayed. ANG II levels were measured by a competitive radioimmunoassay, using the commercially available RIA kit (ED29051, IBL Int., Hamburg, Germany).

**The measurement of plasma and tissue angiotensin 1-7 (ANG 1-7) concentrations**

Plasma and kidney ANG 1-7 levels were measured by a competitive radioimmunoassay using the custom-made RIA kit (Immunotech s.r.o./BeckmanCoulter, Prague, Czech Republic).

*Preparation of plasma and tissue samples* *was similar* as for the determination of ANG II, with a few exceptions. First, 2 ml of plasma was precipitated with 4 ml of ethanol, centrifuged, evaporated and stored. Second, both plasma and kidney samples were purified by SPE, however, a different protocol of SPE was used.

Dried plasma and kidney samples were reconstituted with 4 ml of 50 mM sodium phosphate buffer (pH 7.4) containing 267 mg BSA/l and kept on ice. C18-bonded SPE columns (Bond-Elut^®^C18, Agilent) were preconditioned with mixture of ethanol + distilled-water + 4% acetic acid (83:13:4 by volume; 5 ml), methanol (5 ml), distilled-water (5 ml) and with 4% acetic acid (5 ml). Thereafter, reconstituted samples were applied to pre-washed columns. The columns were sequentially washed with distilled-water (5 ml) and acetone (5 ml). At the end, ANG peptides were eluted from SPE columns with 2x 1ml + 1x 1.5 ml of mixture of ethanol + distilled-water + 4% acetic acid (83:13:4 by volume). The eluates were evaporated to dryness using a vacuum centrifuge. Dried samples were stored at -20°C or lower until assayed.

**Western blot analysis for quantification of kidney protein expression**

The kidney protein expression of AT1 and Mas receptors, ACE and ACE2 were methodically performed as described in detail in our previous studies (3,5). Briefly, kidney cortex was homogenized in 1:3 wt:vol in ice–cold RIPA lysis buffer containing 50 mM Tris–HCl pH 7.4, 150 mM NaCl, 1% NP-40, 0.25% deoxycholic acid, 1 mM EDTA; supplemented with protease inhibitor cocktail (Sigma-Aldrich, St. Louis, MO, USA), kept on ice for 30 min and centrifuged twice - at 10.000 g for 10 min at 4°C and at 20.000 g for 10 min at 4°C. Protein concentration in the supernatant was measured using Pierce BCA protein assay (Thermo Scientific, Waltham, MA, USA). In total 40 μg of protein was separated by sodium dodecyl sulfate polyacrylamide gel electrophoresis (SDS-PAGE) and transferred onto the polyvinyl difluoride (PVDF) membrane in transfer buffer at 100 V for 1.5 hours. Membranes were blocked with 5% non-fat dry milk in TRIS buffered saline with Tween20 (TBS-T) overnight at 4°C. After washing with TBS-T, the membranes were incubated with primary antibodies overnight at 4°C. The antibody dilutions were as follows:

anti-AT1 r., 1:500, Abcam (ab124734; Cambridge, UK);

anti-Mas r., 1:200, Alomone Labs (AAR-013; Jerusalem, Israel);

anti-ACE, 1:500, Abcam (ab222739; Cambridge, UK);

anti-ACE2, 1:100, GeneTex (GTX63044; Irvine, CA, USA);

anti-β-actin, 1:7500, Sigma Aldrich (A5441; MO, USA)

After O/N incubation and washing, the membranes were incubated with horseradish peroxidase-conjugated secondary antibody for 1 hour at room temperature. After last washing, the immunoblots were exposed to SuperSignal West Dura Substrate (Thermo Scientific, Rockford, IL, USA) for chemiluminiscent detection. Relative densitometry was determined using ImageJ software (NIH, Bethesda, MD, USA). All protein data was normalized to the housekeeping protein β-actin.

***Experimental design – SERIES 2***

Rats were subjected to regional myocardial ischemia/reperfusion as described previously, using an open-chest model (6–8). Animals were anesthetized with pentobarbital sodium (60 mg/kg body weight i.p.). A heparinized cannula was placed in the left carotid artery for BP monitoring with a pressure transducer (Gould P23Gb; Gould Instruments Systems, Valley View, Ohio, USA) and the readings were analyzed by our custom-designed software. Rats were ventilated with roomair at 68 strokes/min (tidal volume of 1.2 ml/100 g body weight) using a rodent ventilator (Ugo Basile, Italy). Heart rate was derived from the BP curve. The rectal temperature was maintained between 36.5 and 37.5°C by a servo-controlled heated table throughout the experiment. Left thoracotomy was performed, and a silk suture 6-0 (Chirmax, Czech Republic) was placed loosely around the left anterior descending coronary artery 1–2 mm distal to its origin. After 10min stabilization, regional ischemia was induced by tightening of the suture threaded through a polyethylene tube. Characteristic changes in myocardial color and the incidence of ischemic arrhythmias verified the complete coronary artery occlusion. After a 20min occlusion period, the ligature was released and reperfusion of previously ischemic tissue continued for 3 h.

**Determination of infarct size:** The infarct size and the area at risk were determined as described previously (6,7), by staining with potassium permanganate and 2,3,5-triphenyltetrazolium chloride (Sigma Aldrich, Prague, Czech Republic), respectively. Briefly, hearts were excised and washed with saline via aorta. The area at risk was delineated by perfusion of 5% potassium permanganate after coronary artery reocclusion. Frozen hearts were cut into slices 1mm thick, stained with 1% 2,3,5-triphenyltetrazolium chloride (pH 7.4, 37°C) for 30 min, and fixed in formaldehyde solution. The infarct size and area at risk were determined by computerized planimetry and the infarct size was normalized to the area at risk (infarct size/area at risk). The weight of the LV was determined and the area at risk was normalized to the LV (area at risk/LV). This approach has been validated in many experimental studies and is currently accepted as the ‘gold standard’ for measurement of myocardial infarct size in small animals (9). **Analysis of arrhythmias:** The incidence and severity of ventricular arrhythmias during the 20min ischemic insult and during the first 3 min of reperfusion were assessed according to the recommendation of the Lambeth Conventions (10), as established 25 years ago and is currently accepted as the ‘gold standard’ for the analysis of incidence and severity of arrhythmias. Briefly, premature ventricular complexes (PVCs) occurring as singles, salvos (2 or 3 PVCs) or tachycardia (a run of 4 or more consecutive PVCs) were counted separately. The incidence of ventricular tachycardia and ventricular fibrillation were also determined. Ventricular fibrillation lasting more than 2 min was considered as sustained and animals were not treated to reverse theses abnormalities. The hearts from animals exhibiting sustained ventricular fibrillations were, in accordance with generally accepted approach (9,11), excluded from further evaluation for determination of infarct size. Such hearts are exposed to a tremendous ischemic insult that further alters the infarct size. However, the data were used for analysis of incidence and severity of arrhythmias. The severity of arrhythmias in each group was evaluated by an arrhythmia score according to the incidence of the most severe form of arrhythmia that occurred in each individual heart (heart with single PVCs were given a score of 1, salvos – 2, ventricular tachycardia – 3, reversible ventricular fibrillation – 4, sustained ventricular fibrillation – 5) as described previously in our studies (6,7). This standardized approach for determination of infarct size and arrhythmias enables a comparison of effects of various cardioprotective strategies inside one research group throughout the time, but also with other research groups worldwide (3,4,16,8–15).

**SUPPLEMENTARY FIGURES**


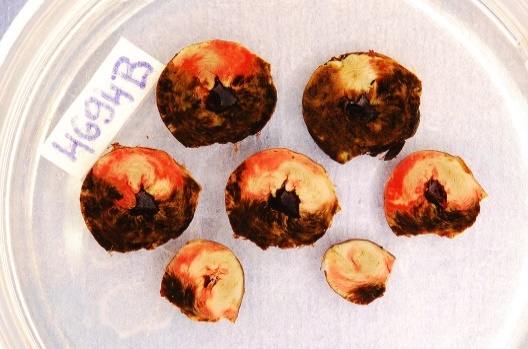

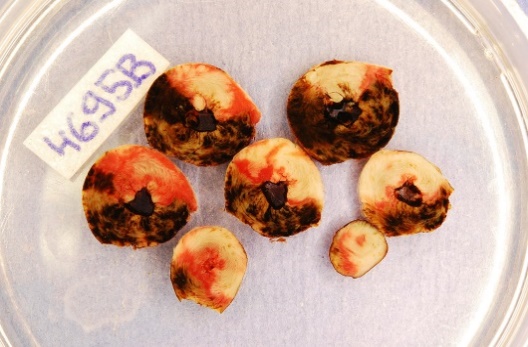

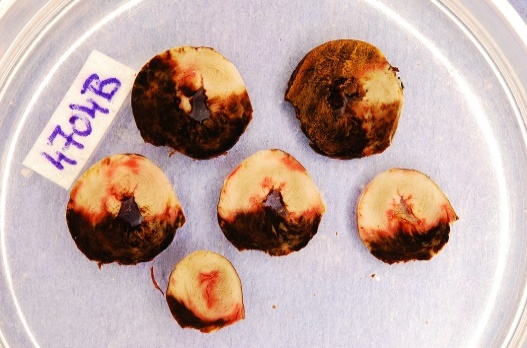

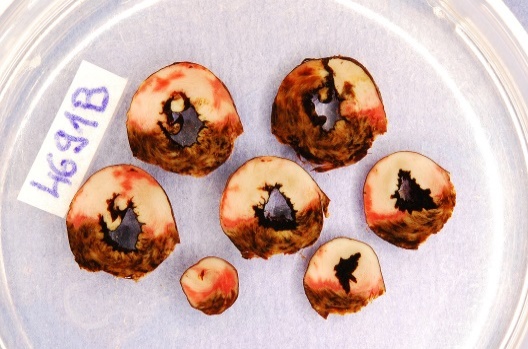


0h I3C (NI)

5d I3C

**Control**

**Control**

**+ losartan**

**+ losartan**

**Supplementary Figure S1.** Typical examples of myocardial infarction induced by 20min coronary artery occlusion and 3h reperfusion in untreated (control) and losartan-treated noninduced (NI) and I3C-induced *Cyp1a1-Ren-2* transgenic rats. Brown color represents normally perfused tissue stained by potassium permanganate. Red area, tetrazolium positive, represents tissue surviving the occlusion, and pale area, tetrazolium negative, is infarcted tissue.

**Supplementary Figure S2**. Representative electrocardiogram recordings showing single premature ventricular complexes (S), double premature ventricular complexes (D), ventricular tachycardia (VT) and ventricular fibrillation (VF) during 20min coronary artery occlusion.

**REFERENCES**

1. Sporková A, Jíchová Š, Husková Z, Kopkan L, Nishiyama A, Hwang SH, et al. Different mechanisms of acute versus long-term antihypertensive effects of soluble epoxide hydrolase inhibition: Studies in Cyp1a1-Ren-2 transgenic rats. Clin Exp Pharmacol Physiol. 2014;41(12):1003–13.

2. Jíchová Š, Kopkan L, Husková Z, Doleželová Š, Neckář J, Kujal P, et al. Epoxyeicosatrienoic acid analog attenuates the development of malignant hypertension, but does not reverse it once established: a study in Cyp1a1-Ren-2 transgenic rats HHS Public Access. J Hypertens. 2016;34(10):2008–25.

3. Husková Z, Kopkan L, Červenková L, Doleželová S, Vaňourková Z, Škaroupková P, et al. Intrarenal alterations of the angiotensin-converting enzyme type 2/angiotensin 1-7 complex of the renin-angiotensin system do not alter the course of malignant hypertension in Cyp1a1-Ren-2 transgenic rats. Clin Exp Pharmacol Physiol. 2016;43(4).

4. Honetschlägerová Z, Husková Z, Vaňourková Z, Vaňourková V, Sporková A, Kramer HJ, et al. Renal mechanisms contributing to the antihypertensive action of soluble epoxide hydrolase inhibition in Ren-2 transgenic rats with inducible hypertension. J Physiol J Physiol. 2011;589(1):207–19.

5. Gawrys O, Husková Z, Baranowska I, Walkowska A, Sadowski J, Kikerlová S, et al. Combined treatment with epoxyeicosatrienoic acid analog and 20-hydroxyeicosatetraenoic acid antagonist provides substantial hypotensive effect in spontaneously hypertensive rats. J Hypertens [Internet]. 2020 Sep 1 [cited 2020 Sep 4];38(9):1802–10. Available from: https://pubmed.ncbi.nlm.nih.gov/32384390/

6. Alánová P, Husková Z, Kopkan L, Sporková A, Jíchová Š, Neckář J, et al. Orally active epoxyeicosatrienoic acid analog does not exhibit antihypertensive and reno- or cardioprotective actions in two-kidney, one-clip Goldblatt hypertensive rats. Vascul Pharmacol [Internet]. 2015 Oct 1 [cited 2020 Jul 10];73:45–56. Available from: http://dx.doi.org/10.1016/j.vph.2015.08.0131537-1891/

7. Neckár J, Kopkan L, Husková Z, Kolár F, Papousek F, Kramer HJ, et al. Inhibition of soluble epoxide hydrolase by cis-4-[4-(3-adamantan-1-yl-ureido)cyclohexyl-oxy]benzoic acid exhibits antihypertensive and cardioprotective actions in transgenic rats with angiotensin II-dependent hypertension NIH Public Access Author Manuscri. Clin Sci. 2012;122(11):513–25.

8. Hrdlička J, Neckář J, Papoušek F, Husková Z, Kikerlová S, Vaňourková Z, et al. Epoxyeicosatrienoic Acid-Based Therapy Attenuates the Progression of Postischemic Heart Failure in Normotensive Sprague-Dawley but Not in Hypertensive Ren-2 Transgenic Rats. Front Pharmacol [Internet]. 2019 Mar 1 [cited 2020 Jul 14];10:159. Available from: /pmc/articles/PMC6406051/?report=abstract

9. Sanada S, Komuro I, Kitakaze M. Pathophysiology of myocardial reperfusion injury: preconditioning, postconditioning, and translational aspects of protective measures. Am J Physiol Hear Circ Physiol [Internet]. 2011;301:1723–41. Available from: www.ajpheart.org

10. Walker MJA, Curtis MJ, Hearse DJ, Campbell RWF, Janse MJ, Yellon DM, et al. The Lambeth Conventions: guidelines for the study of arrhythmias in ischaemia, infarction, and reperfusion. Cardiovasc Res [Internet]. 1988 Jul 1;22(7):447–55. Available from: https://doi.org/10.1093/cvr/22.7.447

11. Ferdinandy P, Schulz R, Baxter GF. Interaction of cardiovascular risk factors with myocardial ischemia/reperfusion injury, preconditioning, and postconditioning [Internet]. Vol. 59, Pharmacological Reviews. American Society for Pharmacology and Experimental Therapeutics; 2007 [cited 2020 Jul 9]. p. 418–58. Available from: http://pharmrev.aspetjournals.org/content/59/4/418

12. Ferdinandy P, Hausenloy DJ, Heusch G, Baxter GF, Schulz R. Interaction of Risk Factors, Comorbidities, and Comedications with Ischemia/Reperfusion Injury and Cardioprotection by Preconditioning, Postconditioning, and Remote Conditioning. Pharmacol Rev Pharmacol Rev [Internet]. 2014;66:1142–74. Available from: http://dx.doi.org/10.1124/pr.113.008300

13. Gross GJ, Hsu A, Pfeiffer AW, Nithipatikom K. Roles of Endothelial Nitric Oxide Synthase (eNOS) and Mitochondrial Permeability Transition Pore (MPTP) in Epoxyeicosatrienoic Acid (EET)-induced Cardioprotection Against Infarction in Intact Rat Hearts. J Mol Cell Cardiol Author Manuscr. 2013;59:20–9.

14. Oni-Orisan A, Alsaleh N, Lee CR, Seubert JM. Epoxyeicosatrienoic acids and cardioprotection: the road to translation. 2014;

15. Ledvényiová-Farkašová V, Bernátová I, Balis P, Puzserova A, Barteková M, Gablovsky I, et al. Effect of crowding stress on tolerance to ischemia–reperfusion injury in young male and female hypertensive rats: Molecular mechanisms. Can J Physiol Pharmacol [Internet]. 2015 Apr 15 [cited 2020 Jul 9];93(9):793–802. Available from: https://pubmed.ncbi.nlm.nih.gov/26317433/

16. Mozaffari MS, Schaffer SW. Effect of Hypertension and Hypertension-Glucose Intolerance on Myocardial Ischemic Injury. Hypertension [Internet]. 2003 Nov [cited 2020 Jul 9];42(5):1042–9. Available from: https://www.ahajournals.org/doi/10.1161/01.HYP.0000095614.91961.40
